# Deprivation and Its Association with Child Health and Nutrition in the Greater Kampala Metropolitan Area of Uganda

**DOI:** 10.1007/s11524-023-00804-0

**Published:** 2024-03-13

**Authors:** Rornald Muhumuza Kananura, Peter Waiswa, Ronald Wasswa, Ties Boerma, Cauane Blumenberg, Abdoulaye Maiga

**Affiliations:** 1 Department of Health Policy Planning and Management, Makerere University School of Public Health, Kampala, Uganda; 2 Centre of Excellence for Maternal and Newborn Health, Makerere University School of Public Health, Kampala, Uganda; 3 University of Manitoba, Winnipeg, Canada; 4 International Center for Equity in Health, Pelotas, Brazil; 5 Causale Consultoria, Pelotas, Brazil; 6 Johns Hopkins University Bloomberg School of Public Health, Baltimore, MD USA

**Keywords:** Slum, Multidimensional deprivation, Child health, Greater Kampala Metropolitan Area, Uganda

## Abstract

African cities are experiencing increasing living standard disparities with limited evidence of intra-urban health disparities. Using data from the 2006–2016 Uganda Demographic and Health Surveys, we employed the UN-Habitat definition to examine slum-like household conditions in the Greater Kampala Metropolitan Area (GKMA). Subsequently, we developed a slum-like severity index and assessed its association with under-5 common morbidities and healthcare access. We also assessed the characteristics of people in slum-like household conditions. We identified five slum-like conditions: substandard housing conditions, limited water access, overcrowding, unclean cooking fuel, and limited toilet access. By 2016, 67% of GKMA households were classified as slum-like conditions, including 31% in severe conditions. Limited toilet access, overcrowding, and limited water access were the main forms of deprivation.

Living in slum-like household conditions correlated with lower education levels, youth status, unprofessional jobs, and marriage. Compared to neighboring Kampala city urban outskirts, Kampala city households had lower slum-like prevalence. Children in GKMA living in slum-like household conditions were more likely to experience diarrhea (moderate: OR = 1.21[95% CI: 1.05–1.39], severe: OR = 1.47 [95% CI: 1.27–1.7]); fever (moderate: OR = 2.67 [95% CI: 1.23–5.8], severe: OR = 3.09 [95% CI: 1.63–5.85]); anemia (moderate: OR = 1.18 [95% CI: 0.88–1.58], severe: OR = 1.44 [95% CI: 1.11–1.86]); and stunting (moderate: OR = 1.23 [95% CI: 1.23–1.25], severe: OR = 1.40 [95% CI: 1.41–1.47]) compared to those living in less slum-like conditions. However, seeking treatment for fever was less likely in slum-like household conditions, and the association of slum-like household conditions with diarrhea was insignificant. These findings underscore the precarious urban living conditions and the need for targeted health interventions addressing the social determinants of health in urban settings.

## Introduction

It is projected that over half of the population in sub-Saharan Africa will be living in urban areas by 2040 [1]. However, the expansion of social public services, such as water and sanitation facilities, in sub-Saharan African cities has not kept pace with the rapid urban population growth [2–4]. This discrepancy is concerning because high population density in cities is often associated with poor housing conditions and inadequate sanitation, which can contribute to the transmission and recurrence of diseases [2, 5–7]. Consequently, the concentration of large numbers of people in cities can lead to negative outcomes, including environmental degradation and the spread of diseases [8]. This aligns with the concept of the “urban penalty,” which suggests that cities concentrate impoverished individuals and expose residents to unhealthy environments, resulting in a disproportionate burden of poor health [9–11].

The urban poor tend to reside in geographically restricted and unsafe areas, such as riverbanks and wetlands [8]. Moreover, due to urban sprawl, individuals living outside metropolitan areas may have limited access to essential social public services, such as reliable water supply and electricity. While the term slum typically refers to a community of multiple households, it is important to recognize that housing conditions can vary significantly within urban settings for each individual household in a particular place of residence [12]. Therefore, analyzing rural-urban disparities in a generalized manner obscures the inequalities that exist within each group [3, 10, 13, 14]. Thus, it is crucial to identify populations living in slum-like conditions regardless of their place of residence. UN-Habitat defines urban slum dwellers as individuals living in housing with one or more of the following characteristics: inadequate drinking water, inadequate sanitation, poor structural quality or durability of housing, overcrowding, and insecure tenure [15]. While these criteria are indeed measures of living conditions, classifying any household with just one of these characteristics as a slum household is arbitrary [16]. For instance, the absence of a local water supply in all households within an urban area does not necessarily mean they should all be considered slum households [16].

While access to services is generally assumed to be better in urban areas, studies have shown that the deprived urban population often faces higher risks of disease and mortality, along with challenges in accessing appropriate healthcare services and interventions [3]. These individuals typically rely on health facilities with inadequate supplies and fewer trained healthcare providers, and they may resort to self-medication by purchasing over-the-counter drugs more frequently than wealthier city dwellers [3]. Additionally, research has documented that the urban poor may share similar characteristics with those living in rural areas, resulting in poor health outcomes [13]. For example, a study in three African countries (Angola, Central African Republic, and Senegal) found high levels of stunting among both urban and rural poor populations, with no significant differences [13].

The increasing number of towns, cities, and urban populations in Uganda presents important research areas with likely relevance for the larger East African and sub-Saharan African regions. The country is experiencing rapid urbanization at a growth rate of 5.2% per year, leading to uncontrolled physical expansion known as urban sprawl, which comes with a high cost for infrastructure and service provision [17]. Unfortunately, due to poor and uncoordinated urban planning and development, Uganda’s cities are struggling to meet the demands for infrastructure and services [17, 18].

In the Greater Kampala Metropolitan Area (GKMA), the combination of urban sprawl and poor urban expansion planning has led to an increase in the number of slum-like households in the neighboring districts including Mukono, Mpigi, and Wakiso. Consequently, relying solely on population density thresholds to define slums would fail to recognize newly formed slums in such districts that have not yet reached the designated population threshold [12]. Moreover, as a result of urban sprawl, some residences built with durable and standard materials are located in areas lacking essential public services such as water. Therefore, categorizing such households as slum-like would be unrealistic [16].

While it is widely acknowledged that slum dwellers typically face inadequate access to social services, there is limited evidence regarding the health of deprived urban children in Uganda and sub-Saharan Africa. Numerous studies examining inequity and inequality tend to concentrate on differentiating between urban and rural areas, often highlighting the better health and nutrition indicators found in urban populations when compared to their rural counterparts. However, this masks the poorer health indicators among more deprived urban dwellers [11]. This situation is partly due to the standard definition of slum dwellers and the limitations of national sample surveys, which often have limited data to analyze differences between slum and non-slum dwellers within urban areas [19–21]. These national surveys, including demographic and health surveys and multi-indicator cluster surveys, are typically used to monitor development indicators in sub-Saharan Africa but have not been analyzed to provide specific insights into intra- and inter-urban residents’ conditions.

This paper utilizes data from three Uganda Demographic and Health Surveys conducted between 2006 and 2016 to investigate urban deprivation and its impact on the health of children under the age of 5 in GKMA. Deprivation is defined as a standard of living or quality of life below that of the majority within a particular city or society [22]. The specific objectives of the study are as follows: (1) determine a measure of slum-like households; (2) assess the prevalence of severe slum-like households; (3) identify the characteristics of individuals living in slum-like conditions; and (4) examine the association between the severity of slum-like conditions and childhood health outcomes.

## Methods

### Data

Our analysis is based on the three most recent Uganda Demographic and Health Surveys (UDHS) conducted in 2006, 2011, and 2016. These surveys are designed to provide national, regional, place of residence (rural and urban), and city (Kampala) estimates [23–25]. For this study, we focused specifically on the Greater Kampala Metropolitan Area, which includes Kampala City and other urban areas within the neighboring districts of Mukono, Wakiso, and Mpigi [17]. To ensure consistency in our analysis, we performed a redistricting of the 2006 and 2011 UDHS data to match the administrative units used in the 2016 survey. This was accomplished through spatial analysis and the use of survey cluster shapefiles. Furthermore, we utilized the Demographic and Health Surveys (DHS) covariate dataset and the Global Human Settlement Model grid (GHS-SMOD) urban–rural population classification data to select only urbanized areas [26]. The GHS-SMOD classification in the DHS dataset categorizes the urban–rural population into three groups: rural cells, urban clusters, and urban centers [26].

To assess the impact of redistricting on the sample size of the Kampala area, we examined any notable differences but found that there were no significant changes (see Appendix Table 3). Additionally, we carefully examined the UDHS questionnaire and determined that all the measures of deprivation used in our study for the three survey years (2006–2016) exhibited a high degree of compatibility, allowing for the comparison of similar indicators (see Appendix Table 4).

### Study Variables and Analysis

#### Measures of Urban Deprivation or Slum

We utilized the UDHS household roster dataset to create a Slum-like Severity Index, which was then linked with other datasets. The items included in the index were selected based on the household characteristics (Appendix 2) commonly used by UN-Habitat to assess the living standards of slum residents [15]. We expanded on the UN-Habitat slum definition [15] by incorporating additional characteristics that are often associated with slum households in Uganda (see Appendix Table 4 and Appendix 2).

To generate a slum-like deprivation score, we conducted a factor analysis that included 11 items to identify domains and their elements. Before performing the factor analysis, we conducted a Kaiser–Meyer–Olkin (KMO) test to assess the adequacy of the sample, as well as a Bartlett test of sphericity to evaluate the suitability of each item for factor analysis [27]. The sample size for the items included in the factor analysis was determined to be adequate (KMO = 69%), indicating a strong partial correlation and the suitability for factor analysis (see Appendix Table 5). The results of Bartlett’s test of sphericity revealed a low probability of the correlation matrix being an identity matrix (*χ*^2^ = 4533, *p* < 0.001), indicating its appropriateness for factor analysis. We identified five factors with eigenvalues greater than or equal to 1 (see Appendix Fig. 3) and individual item scores of at least 0.5, which were then defined as five domains: substandard housing conditions; limited water access; overcrowding; unclean cooking fuel (excluding charcoal), which creates severe indoor air pollution; and limited toilet access (see Appendix Table 6). We created a composite deprivation score by weighting the domains according to the number of items they contained (see Appendix 3).

In contrast to Patel et al. [12], our approach involved using the concept of deprivation, which refers to a lower standard of living or quality of life compared to the majority in a specific society [22]. Based on this concept, we developed the Slum-like Severity Index. While Patel et al. aggregated five items to generate an index that ranged between 0 and 5, with 0 indicating non-slum status and 5 indicating the lack of five basic housing elements considered as the poorest living conditions [12], our study utilized Z-scores to develop our Slum-like Severity Index, taking into account the survey year as follows:

1 $${Z}_{ti}=\frac{{x}_{ti}-{\mu }_{t}}{{\sigma }_{t}}$$

In this equation $, {Z}_{ti}$ is the z-score for household $i$ at time $t;$ ${x}_{ti}$ is the deprivation score for household $i$ at time t; ${\mu }_{t}$ is the mean score at time t; and ${\sigma }_{t}$ is the standard deviation of the deprivation score at time t. We generated the Slum Severity Index by categorizing z-scores as < 0 less slum-like; = 0 moderate (average living conditions) slum-like living conditions; and > 0 severely slum-like living conditions.

#### Deprived Group

To examine the characteristics of children parents or caretakers residing in different household living conditions, we ran a multinomial logistic regression model on our Slum-like Severity Index as a dependent variable controlling for the socio-economic and demographic variables. The socio-economic variables were education levels, type of employment, and wealth status. The demographic variables were age, sex of the head of the household, women’s and men’s marital status, and place of residence.

#### Child Health and Well-being Indicators

We examined six indicators related to child health and nutritional status: stunting and underweight among children under 5 years of age, moderate or severe anemia in children aged 6–59 months, diarrhea and fever in children under 5 years reported by the mother or caretakers in the past 2 weeks, and the utilization of healthcare-seeking services for children with fever in the past 2 weeks (definitions can be found in Appendix Table 7). To investigate the association between the Slum-like Severity Index and child health outcomes (such as common illnesses) and nutritional indicators, we ran either a logistic or a modified poisson regression model, considering child indicators as dependent variables and incorporating the index and place of residence as covariates. A child was classified as having diarrhea or fever if the mother reported that the child experienced either of these conditions within the 2 weeks preceding the interview. Stunting and underweight were determined based on height-for-age z-scores and weight-for-age z-scores that were below 2 standard deviations, respectively. In the case of anemia, we also examined moderate and severe cases and assessed their association with slum status.

## Results

As of 2016, the prevalence of unimproved toilet facilities, unprotected water sources, and incomplete floors was relatively low and similar across the studied area (Appendix Table 8). Approximately three-quarters of households shared a single toilet with five or more other households. Between 2006 and 2016, the time taken (at least 30 min) to access the main water source in Kampala city reduced from 15.7 to 2%, and the availability of water on premises in Kampala city decreased from 71 to 29% (Appendix Table 8). In 2016, the neighboring city outskirts exhibited the highest rates of unprotected water sources, longer travel times to reach a water source, lack of water on premises, substandard household flooring, and substandard household walls structures (Appendix Table 8). Furthermore, during the same period, the percentage of households without a separate place or room for cooking, those accommodating at least four people per room, those with substandard roofing structures, and those with five or more households sharing a toilet were comparable between Kampala city and the neighboring city outskirts.

### Deprivation Score

Over the course of the study period, a significant proportion of households in the GKMA, approximately 97%, exhibited at least one indicator of slum-like conditions. Interestingly, there was no noticeable distinction observed between Kampala city and neighboring outskirtss (Appendix Fig. 4). In 2006, households in Kampala city had an average of 38% of the identified slum-like conditions (slum-like conditions composite score), which decreased to 30% by 2016 (Appendix Table 9). Similarly, the city outskirts demonstrated a relatively consistent level of average slum-like conditions composite score, ranging from approximately 30 to 34% across the UDHS conducted in 2011 and 2016 (Appendix Table 9).

### Slum-Like Living Conditions in GKMA

**Fig. 1:**
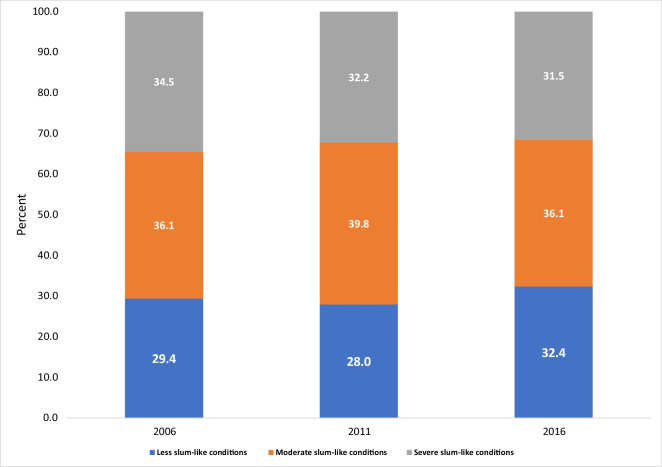
Slum severity index in the Greater Kampala Metropolitan Area

Although there has been a decline in the proportion of households within each category of slum-like conditions over the years, the percentage of households experiencing severe slum-like conditions has remained consistently around 30% throughout the survey period from 2006 to 2016 (Fig. 1).

**Fig. 2:**
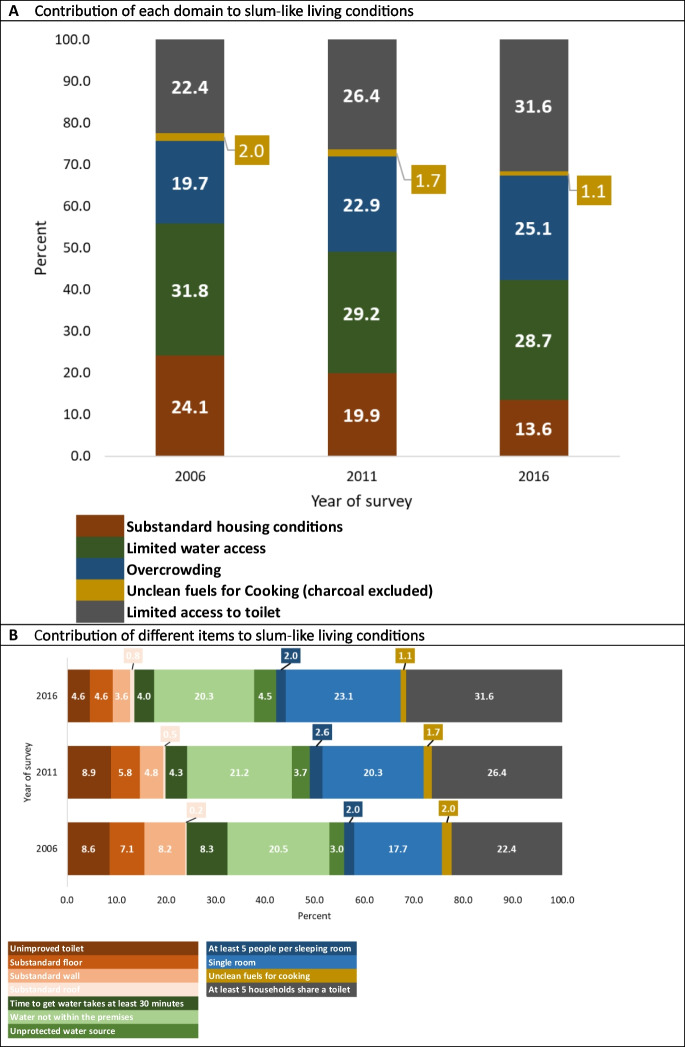
**A** Contribution of each domain and slum measure items to slum-like living conditions. **B** Contribution of different items to slum-like living condition

It is evident that limited access to toilet, limited water access, and overcrowding consistently account for the largest share of slum-like conditions (Fig. 2A). As of 2016, limited toilet access contributed to 32% of slum-like conditions, followed by limited water access at 29% and overcrowding at 25%. In contrast, the use of wood or shrubs for cooking had the least impact on slum-like living conditions over the years.

Analysis of each individual item over the years indicates that sharing a toilet with at least five households had the largest share in contributing to slum-like living conditions, followed by having only one room available and lacking water within the premises (Fig. 2B). 

## Characteristics of People Living in Slum-Like Conditions Based on UDHS Data

Table 10 provides an overview of the characteristics of parents or caregivers of children living in households with moderate and severe slum-like conditions, compared to those in less slum-like household conditions. Individuals in the age group of 20–39 years, both men and women, were more likely to reside in severe slum-like household conditions (20–24: Relative Risk Ratio (RRR) [95%CI] = 2.69 [1.55–4.66], 25–29: RRR [95%CI] = 2.55 [1.48–4.41], 30–34: RRR [95%CI] = 1.81 [1.03–3.16], and 35–39: RRR [95%CI] = 1.84 [1.03–3.28]). Being married was associated with a higher risk of living in households with moderate and severe slum-like conditions (moderate: RRR[95%CI]=1.29[109-154], severe: RRR [95%CI] = 1.33 [1.1–1.6]).

In comparison to households with less slum-like conditions, the likelihood of living in households with moderate and severe slum-like conditions decreased with higher levels of education [higher level in moderate conditions: RRR[95%CI]=0.42[0.33-0.52]; higher level in sever conditions: RRR [95%CI] = 0.16 [0.12–0.21], and secondary level in moderate conditions: RRR[95%CI]=0.76[0.64-0.89]; secondary level in sever conditions: RRR [95%CI] = 0.52 [0.44–0.62]). In another indication of the protective value of education, individuals in professional occupations had a lower likelihood of living in severe slum-like conditions (RRR [95%CI] = 0.63 [0.47–0.83]). Meanwhile, engaging in manual work and service occupations were associated with a higher risk of residing in households with severe slum-like conditions (manual work: RRR [95%CI] = 1.29 [1.03–1.61], and service work: RRR [95%CI] = 1.39 [1.12–1.72]).

Lastly, compared to neighboring city outskirts, households in Kampala city were less likely to exhibit slum-like characteristics (RRR [95%CI] = 0.62 [0.53–0.72]).

### Child Health Outcomes and Their Association with Slum Scores in the GKMA

The study reveals that anemia was the most prevalent health condition among children across all survey periods, followed by stunting, diarrhea, and fever (Appendix Table 11). In 2016, the prevalence of anemia, stunting, fever, and diarrhea was lower in Kampala city compared to neighboring city outskirts (Appendix Table 11). Between 2011 and 2016, the percentage of children with diarrhea in neighboring city outskirts increased from 13.3 to 23% (Appendix Table 11).

Across GKMA, children living in households characterized by moderate slum-like conditions were 21% more likely to experience diarrhea (OR = 1.21, 95% CI = 1.05–1.39), and nearly three times more likely to experience episodes of fever (OR = 2.67, 95% CI = 1.23–5.8), compared to children residing less severe household conditions (Table 1). Children living in households characterized by severely slum-like conditions were 47% more likely to experience diarrhea (OR = 1.47, 95% CI = 1.27–1.170), and three times more likely to experience episodes of fever (OR = 3.09, 95% CI = 1.63–5.85), compared to children residing in less severe slum-like household conditions. The study also found that children in severely and moderately slum-like household conditions had a 40% (OR = 1.40, 95% CI = 1.41–1.47) and 23% (OR = 1.23, 95% CI = 1.23–1.25) higher likelihood of being stunted, respectively, compared to their counterparts in less slum-like conditions (Table 1). Furthermore, children living in households characterized by severe slum-like conditions were 44% more likely to be moderately and severely anemic (OR = 1.44, 95% CI = 1.11–1.86) than those in less slum-like conditions (Table 1). Compared to Kampala city, children in neighboring city outskirts had an increased risk of acute respiratory infections (OR = 3.05, 95% CI = 1.38–6.73) and a lower risk of diarrhea (OR = 0.39, 95% CI = 0.27–0.56) (Table 1).

**Table 1:** Association of child morbidities and nutrition outcomes with slum status in the Greater Kampala Metropolitan Area

	ARI	Fever	Diarrhea	Stunting	Moderate and severe anemia
	RR* [95%CI]	OR** [95%CI]	OR** [95%CI]	OR** [95%CI]	OR** [95%CI]
Variables					
Less slum-like condition (reference)	[-]	[-]	[-]	[-]	[-]
Average slum-like conditions	26.65 [16.13–44.04]	2.67 [1.23–5.8]	1.21 [1.05–1.39]	1.23 [1.23–1.25]	1.18 [0.88–1.58]
Severe slum-like conditions	11.94 [7.19–19.85]	3.09 [1.63–5.85]	1.47 [1.27–1.7]	1.40 [1.41–1.47]	1.44 [1.11–1.86]
Kampala city (reference)	[-]	[-]	[-]	[-]	[-]
Kampala city outskirts	3.05 [1.38–6.73]	2.76 [0.53–14.36]	0.39 [0.27–0.56]	1.94 [0.86–4.4]	0.77 [0.34–1.74]
Less slum-like condition#Kampala city (reference)	[-]	[-]	[-]	[-]	[-]
Average slum-like conditions# Kampala city outskirts	0.28 [0.12–0.64]	0.2 [0.03–1.6]	1.41 [0.92–2.16]	0.47 [0.05–4.02]	1.28 [0.19–8.57]
Severe slum-like#Kampala city outskirts	0.02 [0.01–0.06]	0.44 [0.04–5.41]	3.47 [2.28–5.28]	0.42 [0.19–0.92]	0.86 [0.33–2.2]

*ARI*, acute respiratory infection; *OR*, odds ratio; *RR*, risk ratio.

*Obtained using modified Poisson regression model.

**Based on the logistic regression model.

#Interaction term

### Fever and Diarrhea Medical Treatment and Care-Seeking Behavior for Caregivers to Under-5 Children and Their Association with Slum Scores in GKMA

Between 2006 and 2016, there was a steady increase in the failure to seek child healthcare services in Kampala city. The percentage of individuals who did not seek medical treatment for fever in Kampala city rose from 25% in 2006 to 32% in 2016 (Appendix Table 12). Similarly, the failure to seek medical treatment for diarrhea in Kampala city increased from 11% in 2006 to 25% in 2016 (Appendix Table 12). As of 2016, the overall estimated failure to seek medical treatment for fever and diarrhea in GKMA was 30% and 22%, respectively (Appendix Table 12). Moreover, as of 2016, the failure to seek medical treatment for fever and diarrhea was lower in neighboring city outskirts compared to Kampala city (Appendix Table 12).

Regarding the association between treatment and care-seeking behavior for caregivers of under-5 children with diarrhea and fever, we found that caregivers from severely slum-like households were less likely to seek treatment for fever, although the association was not statistically significant (Table 2). Additionally, we observed an insignificant association for diarrhea care seeking (Table 2).

**Table 2:** Association of treatment seeking with slum status in the Greater Kampala Metropolitan Area

	Fever treatment	Medical treatment for fever*	Diarrhea treatment	Fever treatment sought within 24 h*
Variables	RR**	RR**	RR**	RR**
Less slum-like condition (reference)	[-]	[-]	[-]	[-]
Average slum-like conditions	1.00 [0.98–1.03]	0.98 [0.96–1.01]	1.135 [0.738–1.746]	0.60 [0.49–0.73]
Severe slum-like	0.88 [0.85–0.92]	0.90 [0.88–0.93]	1.138 [0.448–2.894]	0.72 [0.6–0.86]
Kampala city (reference)	[-]	[-]	[-]	[-]
Kampala city outskirts	0.91 [0.79–1.04]	0.93 [0.85–1.02]	0.461 [0.084–2.53]	0.92 [0.59–1.44]
Less slum-like condition#Kampala city (reference)	[-]	[-]	[-]	[-]
Average slum-like conditions#Kampala city outskirts	1.10 [0.96–1.26]	1.10 [1–1.21]	3.135 [0.582–16.888]	0.06 [0.02–0.14]
Severe slum-like#Kampala City outskirts	1.20 [1.03–1.4]	1.16 [1.04–1.29]	4.295 [0.795–23.203]	1.6 [0.91–2.8]

*For only those who sought fever treatment

*OR*, odds ratio; *RR*, risk ratio.

**Obtained using modified Poisson regression model.

#Interaction term

## Discussion

The first aim of the study was to develop a new measure of slum-like living conditions based on the Uganda Demographic and Health Surveys (UDHS) data. In line with the definition of slums provided by UN-Habitat [15], we identified five key domains that capture slum-like conditions, which can be derived from national household surveys: substandard housing conditions, limited water access, overcrowding, unclean cooking fuel, and limited toilet access. In terms of substandard housing conditions and hygiene, indicators such as the absence of improved toilets, roofs, walls, and floors were considered. Limited access to clean water was characterized by longer travel times to water sources and the absence of water within the premises. Overcrowding was measured by households occupying single rooms and having at least five individuals per sleeping room. Furthermore, the use of unclean fuel for cooking and the sharing of a single toilet by at least four households were identified as key indicators of unhealthy cooking materials and limited toilet access, respectively. These conditions have been consistently associated with adverse child health outcomes, the occurrence and recurrence of infections, and accidents [8, 28]. For instance, houses constructed with poor-quality materials such as substandard wood, thatch, and cardboard pose a higher risk of fire accidents [29]. Overcrowding and limited access to water are associated with the transmission of infectious diseases [8, 28].

Based on the UN-Habitat definition of slum conditions, a significant proportion of the population in GKMA, approximately 97%, resided in households characterized by at least one slum-like condition between 2006 and 2016. This definition, while comprehensive, has limitations as it categorizes most low- and lower-middle-income countries as slum areas, making it challenging to capture modest improvements in living conditions. There is a need for more flexible measures that can effectively monitor progress in these countries. It is important to recognize that living standards vary greatly both within and between countries [22], rendering a globally standardized measure less suitable for accurately estimating local situations. Additionally, the concept of deprivation implies a standard of living below that of the majority in a specific context [22], which contradicts the UN-Habitat approach. To address these measurement challenges, we developed the slum-like severity score based on standardized scores, identifying those living in severely deprived conditions as individuals whose standardized scores fell below the average standard of living conditions.

Based on our localized measure, we discovered that as of 2016, households in GKMA had an average slum-like score of 30%. This indicates that the majority of households in this region experience at least 3 out of 10 measures of slum-like living conditions. Moreover, there have been no discernible improvements in these conditions over the years.

Throughout the years, sharing a toilet with at least five households, living in a one-room household, and lacking water within the premises were the primary contributors to slum-like deprivation. At the same time, there has been an increase in the number of households without a separate place or room for cooking. These findings emphasize the prevalent issues related to poor water and sanitation conditions, highlighting the urgent need for interventions that improve water and sanitation infrastructure within urban communities to alleviate slum-like conditions.

Our estimates of the population living in slum-like conditions are in close agreement with the 2014 estimates provided by UN-Habitat [30] and slightly higher than the figures reported in national reports [17]. As of 2016, around 67% of households in GKMA were characterized by at least 30% of slum-like conditions, and there has been no significant change in this percentage over the span of a decade (66% in 2006 and 70% in 2011). Furthermore, approximately 31% of households were classified as having severely slum-like conditions. The likelihood of living in a slum-like household was higher in the neighboring city outskirts within the overall GKMA than within the city of Kampala itself. This trend can be attributed to urban expansion and inadequate urban planning, particularly in these neighboring districts where construction activities are taking place without sufficient public infrastructure and adherence to building regulations [18].

The second objective of our study was to examine the characteristics of caregivers of children living in slum-like conditions. Consistent with previous research [31, 32], we found that lower levels of education, being young in age, being employed in unprofessional jobs, and being married were associated with a higher risk of residing in households with greater slum-like conditions. These findings shed light on the socio-economic factors that influence health outcomes in urban areas. In cities, young individuals often migrate to slum areas where they may experience early pregnancies or marriages [33]. Moreover, while urban settings offer employment opportunities, those living in poverty often face limited skills and end up in low-paying jobs. Consequently, they tend to reside in slum-like households as that is what they can afford, even though these conditions are typically characterized by substandard living conditions.

Lastly, we aimed to investigate the association between slum-like living conditions and child nutrition, common illnesses, and healthcare-seeking behavior in GKMA. We identified disparities in the prevalence of common illnesses and malnutrition among children living in different levels of slum-like conditions. The likelihood of experiencing fever, anemia, diarrhea, and stunting significantly increased with the severity of slum-like conditions. These findings align with existing research that links these conditions to nutritional deficiencies, infections, and poor environmental circumstances [28, 29], which were identified as key indicators of slum-like living conditions in our study. Furthermore, previous studies focusing on intra-urban health disparities have shown that the likelihood of stunting and anemia is higher among children living in poorer urban clusters compared to those in non-poor urban clusters [34–36]. These results are not surprising, as more affluent families generally have greater access to resources that support better childcare practices, thus protecting their children from infections and related health issues.

The residents of slum-like households in GKMA exhibited low treatment and care-seeking behavior when it came to fever among under-5 children. Surprisingly, however, there were no differences in accessing treatment and care for suspected diarrhea based on slum-like status. Various factors, such as maternal education and employment status, have been identified as influential in mothers’ healthcare-seeking behavior. However, the impact of employment status on healthcare-seeking can be complex. In an urban context, women engaged in full-time employment face the challenge of balancing family and work responsibilities, which limits their time to seek healthcare services. This is especially true for those in the informal sector who may have no time or leave days to attend to their sick children. Additionally, caregivers or mothers often seek treatment based on the symptoms of the illness. Our findings indicate the need for child health interventions that focus on preventive and protective measures for all urban residents, which could help to reduce the need to seek out and obtain treatment. However, further studies are required to understand the barriers that prevent access to appropriate healthcare services.

The study has a major limitation in that it only used household-based items to construct the Slum-like Severity Index, overlooking other community context indicators such as population density, uncollected garbage, contaminated water, open sewers, and poor drainage, which are clear indicators of slum areas. However, the items included in the analysis align with the specific criteria outlined by UN-Habitat in its definition of slum or slum-like living conditions. Additionally, the DHS dataset is not specifically designed to provide precise estimates for slum and non-slum dwellers. Nevertheless, by leveraging the DHS GIS data and national administrative GIS boundary data, we were able to reconstruct the DHS cluster/district names, which improved the sample size sufficient to measure slum-like housing conditions. Another limitation is that the reporting of fever and diarrhea in the UDHS relies on self-reporting, which involves recalling past events and self-definition of the severity of conditions. This may introduce some reliability issues in the estimates. However, the use of a reference period of collecting data on events that occurred in the last two weeks prior to the survey helps to minimize recall biases.

In conclusion, our analysis of national household surveys has provided estimates of households experiencing slum-like conditions in the Greater Kampala Metropolitan Area. The study results enhance our understanding of the precarious living conditions in urban areas and offer insights into the characteristics of individuals living in such conditions. The results indicate that a significant proportion of households in the region continue to face poor household living conditions, with little change observed over a decade. The identified indicators linked to slum-like conditions include the sharing of toilets among at least five households, inadequate water access within premises, and residing in single rooms. These conditions have notable implications for childhood health issues, nutrition, and the utilization of healthcare services for children. They also highlight the presence of health inequalities within urban areas. Therefore, efforts should be focused on improving housing quality, ensuring access to clean water and sanitation facilities, and enhancing healthcare services to alleviate the health challenges faced by populations living in slum-like conditions. Furthermore, various individual factors such as age, employment type, education level, and marital status have emerged as key determinants of living in slum-like households. Recognizing these factors is crucial for addressing the social determinants of health in urban settings and tailoring appropriate healthcare services.

Additionally, it is important to conduct larger survey samples to gain a deeper understanding of the living conditions experienced by disadvantaged households in urban areas and their impact on child health and nutrition. By doing so, we can further enhance our knowledge and inform targeted interventions to improve the well-being of these vulnerable populations.

## Figures and Tables

**Fig. 3:**
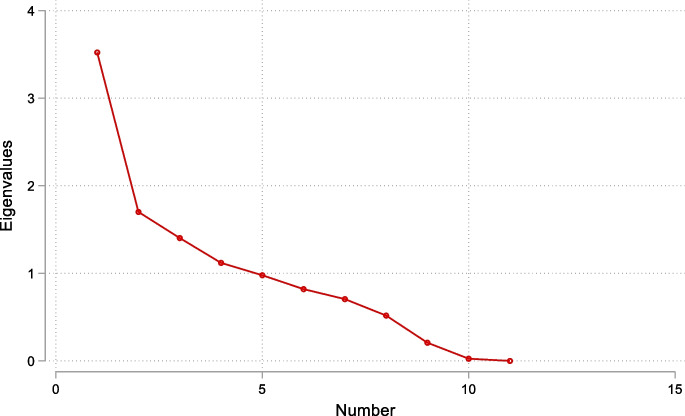
Scree plot representing eigenvalues and number of components in the factor analysis

**Fig. 4:**
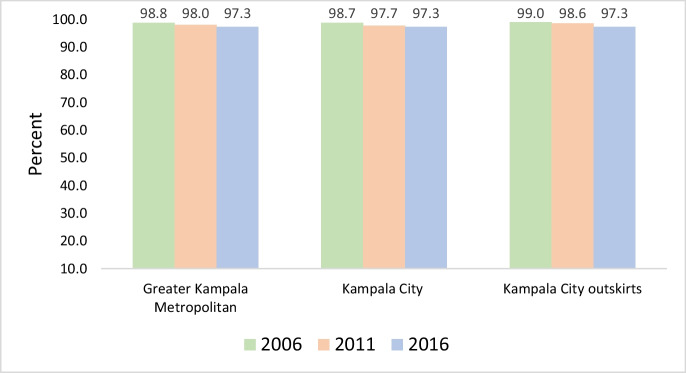
Trend in the proportion of persons with at least one condition of the slum-like environment

## Data Availability

The study utilizes the Uganda Demographic and Health Survey (DHS) data, which is publicly available for free download after registration as a DHS data user.

## Appendix 1

Tables 3, 4, 5, 6, 7, 8, 9, 10, 11 and 12

**Table 3:** District sample size before and after clusters’ reconstruction through GIS

	Before merging					After merging				
	2000	2006	2011	2016	Total	2000	2006	2011	2016	Total
Kampala	NA	485	606	686	1777	143	485	600	697	1925
Mpigi	NA	NA	NA	NA	NA	0	14	55	53	122
Mukono	NA	NA	NA	NA	NA	186	99	125	236	646
Wakiso	NA	NA	NA	NA	NA	22	90	171	404	687
Total	NA	485	606	686	1777	351	688	951	1390	3380

*NA* not available

**Table 4:** Assessment of the data elements

		2000	2006	2011	2016
Sanitation facilities					
hv205	Type of toilet facility	X	x	x	x
hv225	Toilet shared with other hh	X	x	x	x
hv238	Number of HH sharing toilet	NA	x	x	x
hv238	Toilet location				x
Access to water					
hv201	Source of drinking water	X	x	x	x
hv201a	Water interruption	NA	NA	NA	x
hv204	Time to get water	X	X	X	X
hv204	Water on premises	X	x	x	x
hv230b	Location of water at a handwashing place	NA	NA	x	x
Sufficient living area					
hv216	Number of rooms used for sleeping	NA	x	x	x
hv242	Household has a separate place for cooking	NA	x	x	x
v009	Household members	X	x	x	x
hv241	Place where food is cooked	NA	x	x	x
Housing durability					
hv213	Main floor material	X	x	x	x
hv215	Main roof material	X	x	x	x
hv214	Wall material	X	x	x	x
Other					
hv217	Relationship structure	X	x	x	x
hv219	Sex of the hh head	X	x	x	x
hv220	Age of the household head	X	x	x	x
hv226	Type of cooking fuel	X	x	x	x

*NA* not available

**Table 5:** KMO and Bartlett’s test

Kaiser–Meyer–Olkin measure of sampling adequacy		0.688
Bartlett test of sphericity	Approx. chi-square	4533.057
	Degrees of freedom	66
	*p*-value	< 0.001

We performed the Kaiser–Meyer–Olkin (KMO) test for sampling adequacy and Bartlett’s test of sphericity was used to test the null hypothesis that the correlation matrix is an identity matrix [26]. An identity correlation matrix means the variables are unrelated and not ideal for factor analysis. A significant statistical test (≤ 0.05) shows that the correlation matrix is indeed not an identity matrix (rejection of the null hypothesis) [26]

**Table 6:** Factor analysis for identification of slum-like domain measures

Variable	Substandard housing conditions and hygiene	Limited water access	Overcrowding	Cooking with wood-excluding charcoal	Limited access to toilet
Unimproved toilet	0.574				
At least 5 households share a toilet					0.912
Unprotected water source					
Time to get water takes at least 30 min		0.965			
Water not within the premises		0.905			
Substandard roof	0.867				
Substandard wall	0.699				
Substandard floor	0.832				
Unclean fuels for cooking				0.946	
At least 5 people per sleeping room			0.933		
Single room			0.559		

**Table 7:** List of child health indicators

Indicators	Description	Denominator
Underweight	Percentage of children under age 5 who are at least two standard deviations below the median for the reference population for weight-for-age	Children born in the 5 years preceding the survey
Any anemia	Percentage of children aged 6–59 months that have any anemia (< 11.0 g/dl)	Children born 6–59 months preceding the survey
Fever	Percentage of children under age 5 with fever in the 2 weeks preceding the survey	Children born in the 5 years preceding the survey
Diarrhea	Percentage of children under age 5 with diarrhea in the 2 weeks preceding the survey	Children born in the 5 years preceding the survey
Diarrhea and fever treatment access failure	Percentage of children reported to have had diarrhea or fever that never received medical treatment	Children who were reported to have fever or diarrhea but did not get medical treatment

**Table 8:** Trend in measures of households in slum-like living conditions

Living conditions	Kampala city			Other districts within the Greater Kampala Metropolitan			Greater Kampala Metropolitan		
	2006	2011	2016	2006	2011	2016	2006	2011	2016
Unimproved toilet (%)	21.8	21.4	6.3	-	33.2	12.8	-	26.5	10.5
Toilet shared by at least 5 households (%)	68.8	71.8	62.1	-	67.9	71.7	-	70.1	68.3
Unprotected water source (%)	7.0	7.1	1.1	-	18.7	15.7	-	12.1	10.6
Take at least 30 min to the water source (%)	15.7	03.1	1.5	-	24.6	12.8	-	12.4	8.9
Premise without water (%)	71.0	55.5	29.4	-	78.2	58.7	-	65.3	48.5
Substandard floor of household structure (%)	12.9	8.4	3.5	-	26.5	14.2	-	16.2	10.5
Substandard wall of household structure (%)	21.2	11.4	4.8	-	16.7	9.9	-	13.7	8.1
Substandard roof of household structure (%)	0.0	0.4	0.3	-	2.7	6.9	-	1.2	3.3
Households with at least 4 people per room (%)	6.5	5.9	4.7	-	9.4	4.2	-	7.4	4.4
Unclean fuels for coking (%)	6.9	4.7	2.4	-	3.7	2.16	-	4.4	2.2
Have no separate place or room for cooking (%)	19.2	29.5	22.7	-	25.2	28.9	-	28.1	25.4

**Table 9:** *t*-test analysis for the mean slums-like score index between Kampala and other neighbouring district residents

	Variable	Mean composite scores	[95% confidence interval]	
2006	Kampala city	0.38	-	-
	Kampala city outskirts	-	-	-
	Overall	-	-	-
	difference	-	-	-
2011	Kampala city	0.33	0.32	0.33
	Kampala city outskirts	0.34	0.33	0.36
	Overall	0.33	0.32	0.34
	difference	− 0.02	− 0.03	0.00
2016	Kampala city	0.30	0.29	0.31
	Kampala city outskirts	0.30	0.29	0.31
	Overall	0.30	0.29	0.30
	difference	0.00	− 0.01	0.01

**Table 10:** Identified individual characteristics associated with the likelihood of living in severe slum areas through a multinomial logistics regression model

	Moderate slum like		Severe slum like	
Factors	Reference: Less severe slum-like conditions			
	Adj.RRR	95%CI	Adj.RRR	95%CI
Education level (*reference: Lower and no level*)				
Secondary	0.76	0.64–0.89***	0.52	0.44–0.62***
Tertiary or university level	0.42	0.33–0.52***	0.16	0.12–0.21***
Age group (*reference: 45* +*)*^*a*^				
15–19	1.57	0.97–2.52	1.70	0.94–3.07
20–24	1.42	0.91–2.19	2.69	1.55–4.66***
25–29	1.31	0.85–2.03	2.55	1.48–4.41***
30–34	1.17	0.76–1.83	1.81	1.03–3.16*
35–39	1.20	0.76–1.91	1.84	1.03–3.28*
40–44	1.11	0.67–1.83	1.32	0.71–2.46
Marital status (*reference: currently not married*)				
Currently married	1.29	1.09–1.54***	1.33	1.1–1.6***
Household head (*reference: male*)				
Female-headed household	0.83	0.7–0.99*	0.89	0.74–1.08
Occupation (*reference: none*)				
Engaged in a manual occupation	1.11	0.9–1.38	1.29	1.03–1.61**
Engaged in service	1.16	0.95–1.42	1.39	1.12–1.72***
Engaged in a professional occupation	0.79	0.63–0.99*	0.63	0.47–0.83***
District or residence (*reference: Kampala city outskirts*)				
Kampala city	0.81	0.7–0.93***	0.62	0.53–0.72***

^a^Only women aged 15–49 and men aged 15–54; *Adj.RRR*, relative risk ratio; *CI*, confidence interval, ****p* ≤ 0.001, ***p* < 0.01, **p* < 0.05

**Table 11:** Trend in child morbidities and nutrition status

	Kampala city			Kampala city outskirts			Greater Kampala Metropolitan		
	2006	2011	2016	2006	2011	2016	2006	2011	2016
Any anemia	51.7	40.7	49.0	-	62.0	53.9	-	55.7	52.3
Moderate and sever anemia	23.9	17.5	23.9	-	12.7	28.3		16.4	25.4
Fever	18.5	19.5	12.3	-	26.3	22.8	-	24.3	19.4
Diarrhea	23.0	24.5	17.5	-	13.3	23.2	-	16.6	21.4
Underweight	14.7	12.3	3.3	-	7.5	2.2	-	10.6	02.5
Stunting	25.3	29.0	15.7	-	15.1	24.8	-	19.2	21.8

**Table 12:** Trends in healthcare and treatment seeking

	Kampala city			Kampala city outskirts			Greater Kampala Metropolitan		
	2006	2011	2016	2006	2011	2016	2006	2011	2016
Advice sought for fever	83.3	96.2	92.5	-	91.7	87.8	-	94.6	90.0
Received treatment within the first 25 h	-	-	57.9	-	-	58.0	-	-	57.9
No medical treatment for fever	25.2	30.0	32.1	-	31.7	27.3	-	30.6	29.6
No medical treatment for diarrhoea	11.5	12.7	25.2	-	26.1	19.7	-	15.1	22.4
Sought diarrhea treatment	72.89	74.7	70.7	-	69.8	67.2	-	73.8	68.9

## Appendix 2

Description of the Items Used as a Measure of Slum-Like Household Conditions

*Household structure:* Households living in slums usually occupy non-durable dwelling units that expose them to high morbidity and mortality risks. Durable structures are part of the five key components of the agreed definition of the slum [36]. Generally, a housing structure is considered durable when certain strong building materials are used for roofs, walls, and floors [36].

*Overcrowding:* This is a crucial indicator measuring the basic human need for shelter [36]. Reduced space per person is often associated with certain categories of health risks and, therefore, is considered a key criterion for defining the slum [36]. Overcrowding is associated with a low number of square meters per person, high occupancy rates number of persons sharing one room, and a high number of single-room units [36]. Examples of slums worldwide show that dwelling units are often overcrowded with five or more persons sharing a one-room unit used for cooking, sleeping, and other household activities[36]. A house is considered to provide a sufficient living area for the household members if three or fewer people share the same room [36]. In this study, overcrowding was not only based on single rooms but also when there were at least 5 people per sleeping room.

*Access to clean water:* Water is one of the great necessities of human life. Improving access to safe water implies less burden on people and means reducing the global burden of water-related diseases and improving the quality of life. This indicator monitors access to improved water sources based on the assumption that improved sources are likely to provide safe water [36]. The guidelines classify sources of drinking water as improved and unimproved, mainly by using technology to access water: piped water to the dwelling unit or yard, public tap or standpipe, and boreholes are considered improved sources of drinking water, whereas tanker trucks, unprotected wells and springs, and bottled water are considered unimproved sources of water [36]. Additionally, water should be affordable and sufficient quantity should be available without excessive physical effort and time [36]. In this study, poor water access was measured by at least 30 min taken to fetch water and the availability of water within the premises.

*Access to improved sanitation:* Lack of sanitation is a major public health problem that causes disease, sickness, and death. Cholera and diarrhea are usually spread easily through poor hygiene and inadequate sanitation [36]. Poor sanitation risks are greater in slum areas where it is more difficult to avoid contact with waste [36]. Toilet facilities such as sewers or septic tanks, poor-flush latrines, and ventilated improved pit latrines are assumed to be improved, provided they are not public [36]. In addition, the toilet facilities must be correctly constructed, adequately maintained, and not shared by more than two households [36].

*Firewood used for cooking:* The use of firewood for cooking is common among urban poor, including slum dwellers in Uganda [22] and other low and middle-income countries such as India and Bangladesh [37,38]. While the use of charcoal is not considered clean fuel, its use in Uganda is almost universal.

*Electricity not used for lighting:* Urban poor may not have money to pay for electricity bills and thus may opt to use other means of lighting.

*Cooking is done in the house but not in a separate room:* While the urban rich may be cooking inside the house, they usually have a separate room for cooking or a kitchen. However, the urban poor usually cook in their houses and have no separate room for cooking.

## Appendix 3

Slum-Like Household Conditions’ Composite Index Based on 2000–2006 Datasets

The composite index weighting was obtained as follows:

2 $${w}_{i}=\frac{1}{{n}_{d}}$$

3 $${w}_{dij}^{*}=\frac{1}{{n}_{d}}*\frac{1}{{n}_{di}}$$

where ${w}_{i}$ is the weight for $i$ domain, ${n}_{d}$ is the number of domains, ${w}_{dij}^{*}$ is the weight for indicator $i$ in $d$ domain for $j$ individual, and ${n}_{di}$ is the number of $i$ indicators in $d$ domain. Note that $\sum_{j}^{n}{w}_{di}^{*}=1$ and $\sum_{i}^{n}{w}_{di}=1$. Last, we assigned each person slum-deprivation scores according to slum-deprivations in the component indicators. Note that ${w}_{j}$ is the composite slum-deprivation score for individual $j$. The slum-deprivation score of each person is calculated by taking a weighted sum of the number of deprivations so that the deprivation score for each person lies between 0 and 1. The score increases as the number of deprivations of the person increases and reaches its maximum of 1 when the person is deprived in all component indicators. A person, who is not deprived in any indicator, receives a score equal to 0.

The deprivation score is calculated as:

4 $${c}_{j}=\sum_{k=1}^{n}{w}_{dik}^{*}{I}_{k}$$

where ${{\varvec{c}}}_{{\varvec{j}}}$ is individual’s ${\varvec{j}}$ deprivation score, ${{\varvec{w}}}_{{\varvec{d}}{\varvec{i}}}^{*}$ is the weight for indicator ${\varvec{i}}$ in domain ${\varvec{d}}$. ${{\varvec{I}}}_{{\varvec{k}}}=1$ means that the individual is deprived in indicator ${\varvec{k}}$ and ${{\varvec{I}}}_{{\varvec{k}}}=0$ otherwise.

## Appendix 4

Figures 3 and 4
